# New function of gossypol, a natural product of cotton

**DOI:** 10.1186/s42397-022-00135-6

**Published:** 2022-12-01

**Authors:** Jinquan Huang, Xiaoya Chen

**Affiliations:** State Key Laboratory of Plant Molecular Genetics, CAS Center for Excellence in Molecular Plant Sciences, Shanghai Institute of Plant Physiology and Ecology, University of CAS, Chinese Academy of Sciences, Shanghai, 200032 China

Cotton plants are not only the global crops integrating fiber, oil and protein (Hu et al. 2020; Xiao et al. 2017), but also the medicinal plants with potent application values, which were well described in the most complete and comprehensive medical book such as Compendium of Materia Medica and Chinese Materia Medica. Cotton extract has various activities such as stopping bleeding, relieving pain, removing phlegm, and curing chronic bronchitis. Different types of metabolites or defense compounds are produced in cotton plants, including the sesquiterpene aldehyde gossypol, the flavonoids gossypetin and gossypin, and the trisaccharide gossypose (Patel and Patel 2021; Tanyeli et al. 2020; Tian et al. 2018; Kouakou et al. 2009). These metabolites have a variety of pharmacological activities. For example, gossypol was inhibitive to breast cancer (Xiong et al. 2017), ovarian cancer (Qu and Wang 2017), prostate cancer (Akagunduz et al. 2010), and other, gossypose has immunomodulatory effects (Abdel-Latif et al. 2020), and gossypin has bactericidal activities (Chamundeeswari et al. 2007). However, partly due to the enormous interests as an economical crop for textile fiber, vegetable oil and feedstuff, the medicinal value of cotton metabolites has received much less attention, which holds, however, the hope to open up a new way for comprehensive utilization of cotton products.

Recently, Wang and colleagues reported a new function of gossypol: acting as a pan-inhibitor against SARS-CoV-2 and its variants (Wang et al. 2022). Firstly, they found that cotton plants were rarely infected by single-stranded RNA (ssRNA) viruses. It implies that cotton natural products may have antiviral compounds. The investigators took a typical ssRNA virus, SARS-CoV-2, as the model to identify the potential antiviral compounds in cotton plants by combining the methods of molecular docking, biochemical experiments, and anti-SARS-CoV-2 assays. It was showed that gossypol could inhibit the replication of SARS-CoV-2 effectively by targeting RNA-dependent RNA polymerase (RdRp), a core enzyme responsible for viral replication. Moreover, given that RdRp is highly conserved in coronaviruses, it was found that gossypol is also effective against other representative coronaviruses. These findings provide new insight into the prevention and control of coronaviruses.

In the course of evolution and the 5 000 years of domestication (Song et al. 2017), cotton plants have established a very large secondary metabolic network, which can produce and store large amounts of secondary metabolites with special functions. For example, gossypol plays an important role in the process of resisting biological stresses such as pathogen invasion and herbivore attacking of cotton plants (Tian et al. 2016). Moreover, gossypol has shown a wide range of antiviral effects in previous studies. In a previous study, gossypol showed the best activity of anti-Zika virus among the 720 natural products tested (Gao et al. 2019). In addition, gossypol also could effectively inhibit other ssRNA viruses, including plant viruses such as tobacco mosaic virus (Li et al. 2014), zoonotic viruses such as avian influenza virus (Yang et al. 2013), West Nile virus (Lopez-Denman et al. 2018), and Hendra virus (Atkinson et al. 2018). The underlying mechanism has not been fully elucidated until Wang and colleagues published this paper. It is likely that gossypol targets the RdRp of ssRNA viruses of a wide range of hosts from plants to animals.

Although gossypol has a few side effects on humans and animals (Gadelha et al. 2014), it has been used in clinical practice in China, such as in the treatments of functional uterine bleeding, uterine fibroids, menorrhagia and endometriosis (Zhang et al. 2018). The inhibition of coronavirus confers gossypol a promising application, and creating cotton germplasms with high-content gossypol would be a potential focus. At present, the biosynthetic pathway of gossypol has been characterized in depth (Tian et al. 2018; Huang et al. 2020), paving the way to create special cotton varieties of medicinal interests with high-accumulation of specific metabolites or intermediates of the gossypol pathway by regulating its metabolic network through synthetic biology approaches.

## Data Availability

All other data generated or analyzed during this study are included in this published article.
